# Enhanced Adsorption of Cadmium by a Covalent Organic Framework-Modified Biochar in Aqueous Solution

**DOI:** 10.3390/toxics12100717

**Published:** 2024-09-30

**Authors:** Yanwei Hou, Shanna Lin, Jiajun Fan, Youchi Zhang, Guohua Jing, Chao Cai

**Affiliations:** 1 College of Chemical Engineering, Huaqiao University, Xiamen 361021, China; houyw@hqu.edu.cn; 2 Key Lab of Urban Environment and Health, Institute of Urban Environment, Chinese Academy of Sciences, Xiamen 361021, China; snlin@iue.ac.cn (S.L.); jjfan@iue.ac.cn (J.F.); yczhang@iue.ac.cn (Y.Z.)

**Keywords:** COF-modified biochar, rice husk, cadmium, adsorption

## Abstract

In the environmental field, the advancement of new high-efficiency heavy metal adsorption materials remains a continuous research focus. A novel composite, covalent organic framework-modified biochar (RH-COF), was fabricated via an in-situ polymerization approach in this study. The COF-modified biochar was characterized by elemental analysis, BET analysis, SEM, FT-IR, and XPS. The nitrogen and oxygen content in the modified material increased significantly from 0.96% and 15.50% to 5.40% and 24.08%, respectively, indicating the addition of a substantial number of nitrogen- and oxygen-containing functional groups to the RH-COF surface, thereby enhancing its adsorption capacity for Cd from 4.20 mg g^−1^ to 58.62 mg g^−1^, representing an approximately fourteen-fold increase. Both the pseudo-second-order model and the Langmuir model were suitable for describing the kinetics and isotherms of Cd^2+^ adsorption onto RH-COF. The adsorption performance of Cd^2+^ by RH-COF showed minimal sensitivity to pH values between 4.0 and 8.0, but could be slightly influenced by ionic strength. Mechanistic analysis showed that the Cd^2+^ adsorption on RH-COF was dominated by surface complexation and chelation, alongside electrostatic adsorption, surface precipitation, and Cπ–cation interactions. Overall, these findings suggest that the synthesis of COF-biochar composite may serve as a promising remediation strategy while providing scientific support for applying COF in environmental materials.

## 1. Introduction

Biochar has been an extremely hot scientific subject over the past two decades, and is regarded as a green environmental material due to its wide availability, facile preparation, low risk of secondary pollution, and low cost [1,2,3]. The application of biochar in the environment can bring numerous benefits, such as enhancing crop yield and soil health, reducing greenhouse gas emissions, reutilizing waste, and alleviating climate change. Owing to its favorable pore structure and abundant surface functional group structure, biochar, as an excellent alternative adsorbent, has been extensively studied in terms of its effect and mechanism for removing heavy metals from both soil and water [4,5]. Technology based on biochar has emerged as one of the highly potential and promising tools for environmental remediation.

Biochar can be derived from numerous agricultural wastes, such as manure, corn stalk, rice straw, rice husk, and bean stalk. Despite the similar appearance of different biochars, their behaviors vary significantly due to the diverse physicochemical properties of biochars, which are profoundly influenced by the type of feed stock [6]. Consequently, some biochars exhibit excellent adsorption performance, while others do not. Generally speaking, biochars produced from poultry manure possess a higher metal adsorption capacity compared to those derived from plants [7,8,9]. Xu et al. reported that biochar originated from dairy manure demonstrated superior performance to rice husk biochar, which has a low surface area and limited functional groups in removing Cu^2+^, Cd^2+^, Zn^2+^, and Pb^2+^ from water [10]. However, the application of these biochars in heavy metal removal is largely hindered by their poor adsorption performance. Cd is a toxic heavy metal element that poses a significant risk to human health. Epidemiological evidence suggests that occupational and environmental cadmium exposure may be associated with many types of cancer, including breast, lung, prostate, nasopharyngeal, pancreatic, and kidney cancers [11]. Therefore, it is essential for these biochars to be modified to enhance their metal adsorption ability.

Consequently, diverse modification approaches of biochars, such as acidification, alkalization, amination, magnetic modification, metal ion incorporation, oxidation using oxidizing agents and surfactant modification, have been developed to obtain specific advantages for numerous particular applications. These approaches may dramatically alter the physicochemical properties of biochars and significantly improve their capacity for adsorbing heavy metals [12]. Chen et al. fabricated sulfur modified wheat straw biochar, which exhibited a superior adsorption capacity for Cd^2+^ from aqueous solutions compared to the original biochar [13]. Fan et al. prepared thiol-modified rice straw biochar with maximum adsorption capacities of 45.1 mg g^−1^ for Cd^2+^ and 61.4 mg g^−1^ for Pb^2+^ [14]. Lee and Shin modified three distinct types of biochar (rice husk, wood chip, and mixture) through five different methods (acid, alkaline, oxidic, manganese oxide, and iron oxide). The results indicated that MnOx-modified biochar achieved the highest adsorption capacities, which increased the adsorption capacity of Cd^2+^ from 3 mg g^−1^ to 10 mg g^−1^ [15]. To date, producing modified biochar material has emerged as an important practice for expanding the environmental applications of biochar.

Covalent organic frameworks (COFs) represent an emerging category of porous substances with pre-designable architectures [16]. COF materials possess numerous specific properties, such as elevated thermal/chemical stability, substantial porosity and surface area, as well as uniform and adjustable pore sizes, endowing them with potential as remediation substances [17,18]. Introducing a covalent organic framework (COF) to the outermost layer of the adsorbent material would generate more oxygen- and nitrogen-containing organic groups, which might enhance the material’s adsorption capacity for target metal ions [19]. The COF-modified material would offer superior porosity, high stability, novel synergistic properties, large specific surface area, high density of nitrogen- and oxygen-containing functional groups, high crystallinity, and novel forms for environmental applications [20,21,22], facilitating the adsorption of metal ions. Many COF-modified materials have demonstrated significant potential in removing heavy metal species. For example, Sun et al. fabricated a COF-S-SH material through post-synthesis modification for Hg^2+^ removal [23]. Jiang et al. prepared an EDTA@COF for the removal of heavy metal ions [24]. Yang et al. made an EB-COF:Br material as an adsorbent for As (V) [25]. Li et al. modified COF on the absorbent material and augmented the adsorption capacity of Cu^2+^ compared with the raw material [26]. Consequently, it is anticipated to enhance the adsorption capacity of COF-modified biochar composites, yet few studies have explored the adsorption performance of this composite.

In this study, a novel composite, COF-modified biochar derived from rice husk, was fabricated. The properties of COF-modified biochar were characterized using modern chemical analysis approaches, and a series of adsorption experiments was carried out to investigate the adsorption performance and mechanism of COF-modified biochar on Cd^2+^, which was selected as a representative heavy metal in environmental media. The outcomes of this study will offer scientific support for the development of efficient adsorbents for the remediation of cadmium-contaminated water.

## 2. Materials and Methods

### 2.1. Chemicals and Reagents

All reagents used in this study were analytical grade and used as received. Ethylene glycol, ammonium hydroxide, N, N-dimethylacetamide (DMAC), dioxane, acetic acid solution, methanol, acetone, tetrahydrofuran, sodium nitrate, disodium hydrogen phosphate, lead nitrate, copper nitrate hydrate, calcium nitrate, magnesium nitrate, and sodium bicarbonate were purchased from Sinopharm Reagent Co., Ltd. (Shanghai, China). Cadmium nitrate and sodium humate were purchased from Aladdin Reagent Co., Ltd. (Shanghai, China). The 2,4,6-trihydroxy-1,3,5-benzenetricarboxaldehyde (Tp) and 4,4′-diamino-[1,1′-biphenyl]-2,2′-dicarboxylic acid (DBd) were purchased from Chinese Academy of Sciences-Yanshen Technology Co., Ltd. (Changchun, China).

### 2.2. Preparation of Materials

Rice husk biochar (RH) was prepared from air-dried rice husk. Rice husk was pyrolyzed at 500 °C for 2 h under oxygen-limited conditions in a muffle furnace (KSL, Kejing Inc., Hefei, China). The RH was ground to pass through a 0.15 mm sieve and then thoroughly mixed for further application.

COF was solvothermally synthesized by the co-condensation of 2,4,6-trihydroxy-1,3,5-benzenetricarboxaldehyde (Tp) and 4,4′-diamino-[1,1′-biphenyl]-2,2′-dicarboxylic acid (DBd), with acetic acid serving as a catalyst. Briefly, 1.60 mmol of Tp and 2.40 mmol of DBd in 120 mL N, N-dimethylacetamide (DMAC)/dioxane (1:11) and 10 mL acetic acid solution (6 mol L^−1^) were mixed in a 200 mL Teflon-tube under ultrasonication. The mixture was subsequently transferred into a 200 mL Teflon-lined autoclave for solvothermal reaction at 120 °C for 3 days. After the reaction, the precipitate was filtered, washed several times with dioxane, methanol, acetone and tetrahydrofuran (THF), and dried at 40 °C for 12 h in a vacuum. The COF was ground to pass through a 0.15 mm sieve and then mixed thoroughly for further experiments.

RH (2.00 g), serving as a raw material, was added to 80 mL ethylene glycol under ultrasonication. Subsequently, an additional 6 mL of ammonium hydroxide was introduced, and the resulting mixed solution was transferred to a Teflon-lined autoclave for solvothermal reaction at 180 °C for 10 h. After the reaction was concluded, the precipitate was filtered, washed repeatedly with distilled water, and dried in a vacuum at 40 °C for 12 h. After pretreatment, the RH was ground to pass through a 0.15 mm sieve and then mixed thoroughly for further application.

The synthesis process of the COF-modified rice husk biochar composite (RH-COF) was identical to that of COF, except that 0.80 g of pretreated RH was added to the mixed solution prior to ultrasonication.

### 2.3. Characterization of Materials

The pH value of the material was determined by weighing a 0.3 g sample and placing it in a 15 mL centrifuge tube, adding 6 mL ultra-pure water, mixing it well, and oscillating for 1 h at 180 r·min^−1^ at 25 °C. Subsequently, the pH value of the sample solution was determined using a pH meter (STARTER 3100/F, Ohaus Inc., Changzhou, China) [14]. The contents of C, N, O, and H in the samples were quantified using a Vario Max CHNS-O-Cl Analyser (Elementar Analysysteme GmbH, Hanau, Germany) [27]. The structural characteristics and elemental composition of the samples were examined via field emission scanning electron microscopy (FE-SEM) (S-4800, Hitachi, Tokyo, Japan) [28,29]. The specific surface area and pore size distribution of the samples were determined with an ASAP 2020 M+C specific surface analyser (Micromeritics, GA, Atlanta, USA) and analyzed by the Brunauer–Emmett–Teller (BET) and Barret–Joyner–Halenda (BJH) methods with N_2_ adsorption isotherms, respectively. The functional groups of the samples were identified utilizing Fourier transform infrared spectroscopy (FT-IR, Nicolet iS10, Thermo, Waltham, MA, USA). The surface element analysis was performed by X-ray photoelectron spectroscopy (Axis Supra, Kratos, Tokyo, Japan) with an Al Kα source (hv = 1486.6 eV) [14]. The surface charge properties of the samples were investigated by zeta potential measurements at varying equilibrium pH values using Zeta PALS (Malvern, Malvern City, UK) [30].

### 2.4. Batch Adsorption Experiments

The adsorption performance of Cd^2+^ by RH and RH-COF was investigated by batch adsorption experiments in a 15 mL polyethylene centrifuge tube. The Cd(NO_3_)_2_ solution in an electrolyte background of 0.01 mol L^−1^ NaNO_3_ was mixed with either RH or RH-COF at a dosage of 2 g L^−1^. The pH of the Cd(NO_3_)_2_ solution was adjusted to the desired values using 1 mol L^−1^ HNO_3_ and 1 mol L^−1^ NaOH.

The Cd^2+^ adsorption kinetics experiments were carried out with 0.20 g of either RH or RH-COF in 100 mL solution containing either 100 or 250 mg L^−1^ Cd^2+^ (pH = 5) in a series of 250 mL glass conical flasks. The samples were periodically withdrawn (0–48 h) and filtered through a 0.45 μm PES water filtration membrane (Keyilong Lab Equipment Co., Ltd., Tianjin, China). The concentration of Cd^2+^ in the filtrate was determined by an inductively coupled plasma optical emission spectrometer (ICP-OES, Optima 7000DV, PerkinElmer, Waltham, MA, USA) after dilution with 2% HNO_3_ solution. Both pseudo-first-order and pseudo-second-order kinetic models were employed to analyze the kinetic parameters.

The Cd^2+^ adsorption isotherms were established by introducing 0.01 g of RH or RH-COF into 5 mL of Cd^2+^ solutions with varying concentrations in 15 mL polyethylene centrifuge tubes. Cd^2+^ concentrations (pH = 5) ranged from 5 to 700 mg L^−1^. The mixture was agitated at a speed of 150 rpm at 25 ± 0.5 °C for 48 h, and then the suspension was filtered through a 0.45 μm PES water filtration membrane to analyze the residual Cd^2+^ concentrations. The adsorption isotherm parameters were derived using both Langmuir and Freundlich models.

The influence of pH on the adsorption of Cd^2+^ was assessed across a range from pH 2.0 to 8.0 with initial pH adjustments using either 1 mol L^−1^ HNO_3_ or NaOH solutions. Additionally, the effect of ionic strength on the adsorption of Cd^2+^ was evaluated by varying NaNO_3_ concentrations between 0.001 and 0.100 mol L^−1^. Furthermore, the influence of H_2_PO_4_^2−^, HCO_3_^−^, Cu^2+^, Pb^2+^, Ca^2+^, Mg^2+^, and HA on the adsorption of Cd^2+^ were investigated by supplementary additions of H_2_PO_4_^−^ (10^−4^ mol L^−1^), HCO_3_^−^ (10^−3^ mol L^−1^), Cu^2+^ (100 mg L^−1^), Pb^2+^ (100 mg L^−1^), Ca^2+^ (10^−2^ mol L^−1^), Mg^2+^ (10^−2^ mol L^−1^), and HA (10^−3^ mol L^−1^) with 250 mg L^−1^ Cd^2+^ solution (pH = 5), respectively. All adsorption experiments were conducted in triplicate.

### 2.5. Data Processing and Statistical Analysis

All data were presented as the means plus or minus standard deviation. Differences among treatments were examined using one-way analysis of variance (ANOVA). Data processing was performed using Origin2017 (OriginLab Corporation, Northampton, MA, USA), while the statistical analysis was conducted using IBM SPSS Statistics 23 software (SPSS Inc., IBM, Armonk, New York, NY, USA).

## 3. Results and Discussion

### 3.1. Physiochemical Properties of Materials

**Figure 1:**
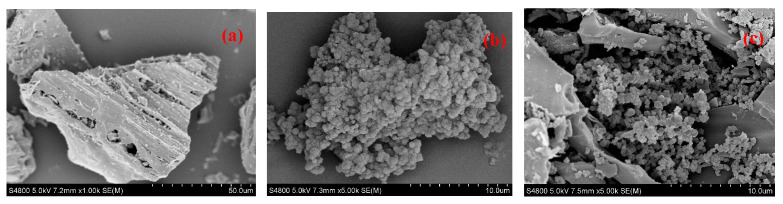
SEM micrographs of RH (**a**) at 1000× magnification, COF (**b**), and RH-COF (**c**) at 5000× magnification.

Field emission scanning electron microscopy (FE-SEM) images of RH, COF, and RH-COF are presented in Figure 1. The RH sample exhibited a rough surface with an irregular porous structure and some attached particles, while a spherical COF structure was observed. The SEM image of the RH-COF composite demonstrated that COF had successfully adhered to both the surface and pores of the composite.

The physicochemical properties of RH, COF, and RH-COF are summarized in Table 1. The elemental analysis indicated that a significant alteration in the composition of RH following modification. The nitrogen content in RH-COF was elevated compared to that in RH, increasing from 0.96% to 5.40%, thereby suggesting the successful modification of RH with COF.

Furthermore, the pH of RH-COF was lower than that of RH (pH 9.67) as shown in Table 1. The pH level of biochar is primarily influenced by its surface functional groups [31]. The observed decrease in pH for RH-COF can be partially attributed to the protonation or deprotonation reaction occurring on the surface of the composite, which might affect its adsorption performance during the application [27].

**Figure 2:**
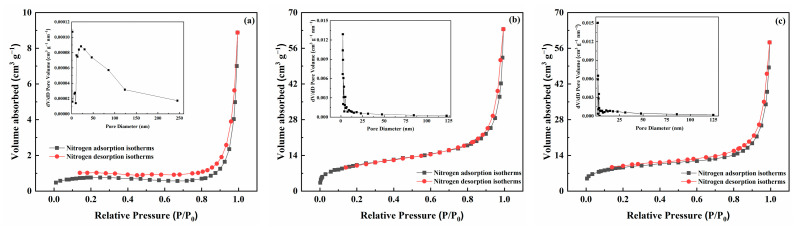
Nitrogen adsorption/desorption isotherms and pore size distributions of RH (**a**), COF (**b**), and RH-COF (**c**).

The nitrogen adsorption/desorption isotherms and corresponding pore size distribution curves for RH, COF, and RH-COF are presented in Figure 2. The nitrogen adsorption/desorption isotherms of RH, COF, and RH-COF exhibited a characteristic IV-type isotherm with an H3 hysteresis loop, indicating the presence of mesopores in these materials (Figure 2) [32]. Moreover, the nitrogen adsorption isotherm demonstrated that following modification RH-COF possessed greater abundance of mesopores compared to RH (Figure 2a,c).

In terms of the surface properties, post-modification analysis demonstrated an increase in both the surface area and average pore volume of RH-COF. Specifically, the surface area of RH-COF increased from 2.70 m^2^ g^−1^ to 33.41 m^2^ g^−1^, however, the pore diameter of RH-COF decreased from 9.24 nm to 6.30 nm due to infilling by COF with a large specific surface area and pore volume (Table 1). The abundant pores facilitate not only the adsorption of solute onto the outer surface of RH-COF but also their diffusion into the pore channels where they can bind to the activated adsorption sites on the inner surface. Notably, since the pore diameter of RH-COF exceeds that of the cadmium at approximately 0.19 nm, it exhibits distinctive adsorption performance towards Cd^2+^.

**Figure 3:**
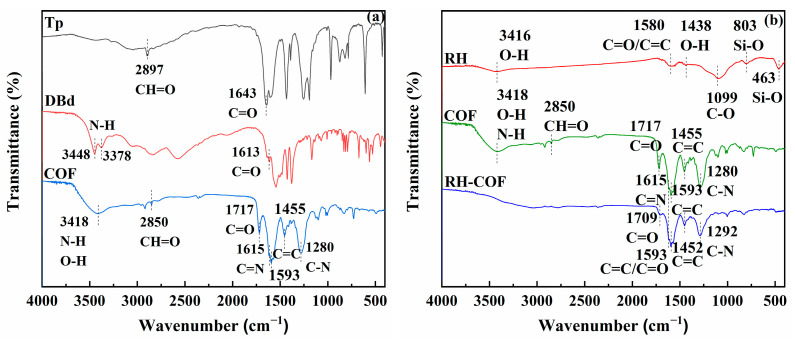
Comparison of the FT-IR spectra of Tp, DBd and COF (**a**), Comparison of the FT-IR spectra of RH, COF and RH-COF (**b**).

The analysis of Fourier transform infrared (FT-IR) spectroscopy was carried out to elucidate the chemical changes of the functional groups on the adsorbent surface. The FT-IR spectrum of RH (Figure 3) presented distinct peaks corresponding to O-H (3416 cm^−1^ and 1438 cm^−1^), C=O/C-C (1580 cm^−1^), C-O (1099 cm^−1^), and Si-O (803 cm^−1^ and 463 cm^−1^) [33,34,35,36,37], indicating the presence of carboxyl and hydroxyl functional groups on the surface of RH [28,38]. In contrast, the FT-IR spectrum of COF presented obvious peaks for N-H/O-H (3418 cm^−1^), CH=O (2850 cm^−1^), C=O (1717 cm^−1^), C=C (1455 cm^−1^ and 1593 cm^−1^), as well as C-N (1280 cm^−1^) [33,36,39,40]. Notably, the FT-IR spectrum of RH-COF presented minimal variation in the composition of the functional groups compared to either COF or RH. Specifically, the peaks for O-H/N-H (3416 cm^−1^ and 3418 cm^−1^) and CH=O (2850 cm^−1^) were absent, while a significant reduction in the peak intensity for C=O (1717 cm^−1^) was observed along with a shift to a new position at 1709 cm^−1^. Additionally, the new peaks corresponding to C=N (1615 cm^−1^), C=C (1452 cm^−1^ and 1593 cm^−1^), and C-N (1292 cm^−1^) were identified in the FT-IR spectrum of RH-COF [39,40,41,42,43]. Therefore, the introduction of nitrogen-containing functional groups (C-N/C=N) and carbon-containing functional groups (C=C/C=O) in RH-COF demonstrated that successful loading of COF on RH had occurred. These functional groups are intrinsically linked to the materials’ physical and chemical properties, and they can influence adsorption capacity.

### 3.2. Adsorption Behaviour of Materials for Cd^2+^

#### 3.2.1. Adsorption Kinetics

**Figure 4:**
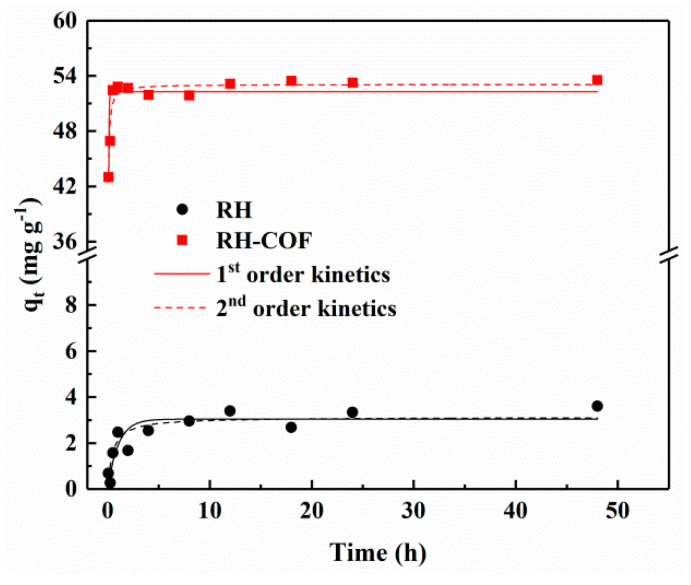
Adsorption kinetics of Cd^2+^ on RH and RH-COF (initial concentration of Cd^2+^: 100 mg L^−1^ (RH) and 250 mg L^−1^ (RH-COF), adsorbent dosage: 2.0 g L^−1^, pH 5).

Adsorption kinetics are crucial for estimating the rate of adsorption and offering valuable insights into the adsorption mechanism. The adsorption kinetics of Cd^2+^ on RH and RH-COF were examined, and the results are shown in Figure 4. The adsorption amount of Cd^2+^ on RH increased gradually, reaching adsorption equilibrium after approximately 2 h. In contrast, the initial adsorption of Cd^2+^ on RH-COF was rapid, achieving equilibrium within 0.5 h, indicating a significant enhancement in the adsorption rate following modification.

To elucidate the adsorption mechanisms, the adsorption kinetics data were fitted to both pseudo-first-order and pseudo-second-order kinetic models. Detailed information on these adsorption kinetics and fitting parameters can be found in the Supplementary Information (Table S1). For both RH and RH-COF, the coefficient of determination (R^2^) for the pseudo-second-order kinetic model (0.92 and 0.90) surpassed that for the pseudo-first-order kinetic model (0.77 and 0.69). This suggests that the adsorption of Cd^2+^ by both materials is more accurately described by the pseudo-second-order kinetic model than by the pseudo-first-order kinetic model, implying that the adsorption predominately governs this process. The process of the pseudo-second-order model can be divided into two distinct kinetic phases. In the initial stage of adsorption, due to the existence of a large number of adsorption sites on the surface of the adsorbent, the adsorption rate was fast. The K_2_ value of RH-COF for Cd^2+^ exceeded that of RH (Table S1), suggesting a faster adsorption of Cd^2+^ on RH-COF compared to its counterpart. However, as time progressed and available adsorption sites were gradually filled, there was a corresponding decline in adsorption rate until the adsorption equilibrium was reached where the difference between their adsorption rates diminished.

#### 3.2.2. Adsorption Isotherms

**Figure 5:**
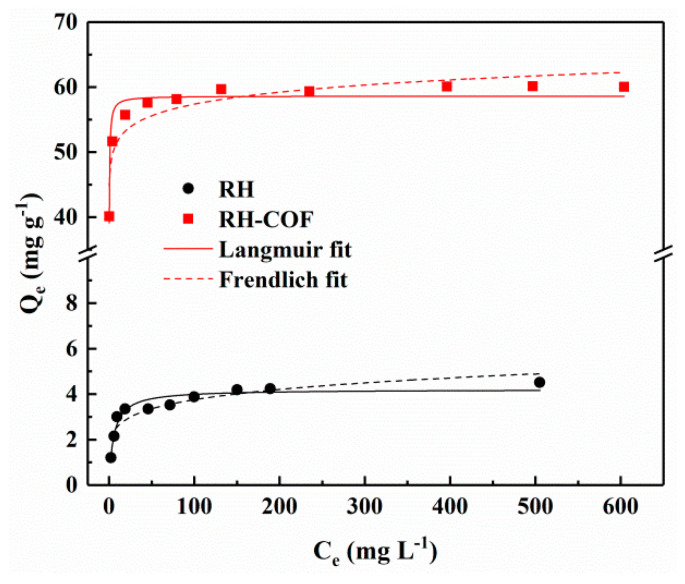
Adsorption isotherms of Cd^2+^on RH and RH-COF (adsorbent dosage: 2.0 g L^−1^, pH 5, contact time: 48 h).

The influence of varying initial Cd^2+^ concentrations on the adsorption isotherms of Cd^2+^ by RH and RH-COF was investigated, with results presented in Figure 5. Comprehensive details regarding the adsorption isotherms and the model parameters fitted using the Langmuir and Freundlich models are available in the Supplementary Information (Table S2). The maximum adsorption capacity of RH-COF for Cd^2+^ surpassed that of RH. As indicated in Table S2, the Langmuir adsorption capacity of RH-COF for Cd^2+^ reached 58.62 mg g^−1^, nearly 14 times greater than that of RH (4.20 mg g^−1^). This finding indicates a significant enhancement in the adsorption capacity of RH for Cd^2+^ following COF modification. Moreover, when compared to previously reported modified biochar adsorbents and other biocarbon sources, RH-COF exhibited a competitive adsorption capacity for Cd^2+^ (Table 2), highlighting distinct advantages associated with COF-modified biochar. According to Table S2, the experimental data for both RH and RH-COF were more accurate by the Langmuir model (R^2^ = 0.92 and 0.90, respectively) than by the Freundlich model (R^2^ = 0.83 and 0.84, respectively). The results imply that metal ion adsorption occurred at a homogeneous surface by monolayer sorption [44].

#### 3.2.3. Effect of Environmental Factors on the Adsorption of Cd^2+^

**Figure 6:**
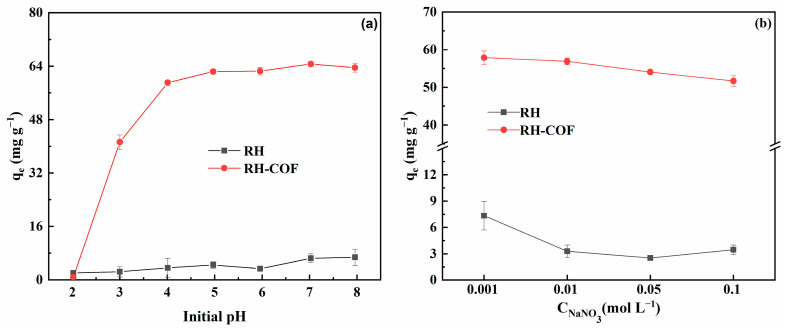
Effect of pH (**a**) and ionic strength (**b**) on the adsorption of Cd^2+^ by RH and RH-COF (initial concentration of Cd^2+^: 250 mg L^−1^, adsorbent dosage: 2.0 g L^−1^, contact time: 48 h).

The pH plays a crucial role in the adsorption process, influencing not only the protonation degree of the functional groups on the adsorbent surface but also the chemical speciation of Cd in the solution [50,51]. This study investigated the effect of an initial pH range from 2.0 to 8.0 on the adsorption of Cd^2+^ by RH and RH-COF, with results illustrated in Figure 6a. As pH increased, the amount of Cd^2+^ adsorption on RH gradually increased at a slow rate. In contrast, the adsorption capacity of RH-COF for Cd^2+^ exhibited a sharp increase as pH transitioned from 2.0 to 4.0, followed by a gradual rise that stabilized beyond pH 5.0. At a pH of 2.0, both RH-COF and RH displayed similar and notably low adsorption capacities for Cd^2+^. However, from pH values ranging between 3.0 and 8.0, the adsorption capacity of RH-COF was significantly higher than that of RH in this study. The influence of pH on the adsorption process may be attributed to the electrostatic interactions and competition between H^+^ and Cd^2+^. The point-of-zero charge (pHpzc) values for RH and RH-COF were found to be 2.49 and 2.84, respectively (Figure S1). When the pH falls below its respective pHpzc, the surface becomes positively charged with an abundance of available H^+^ occupying potential adsorption sites; consequently, these H^+^ hinder the binding interactions between Cd^2+^ and adsorbents. As pH increases, the deprotonation on the adsorbent surface becomes more pronounced, while the competition for binding sites between Cd^2+^ and H^+^ decreases, resulting in more available binding sites which facilitate electrostatic interactions leading to enhanced coupling with both RH and RH-COF by Cd^2+^. When the pH value exceeds 3.0, the adsorption capacity of Cd^2+^ by RH-COF significantly surpasses that of RH, a phenomenon attributed to successful introduction of COF with a high specific surface area and pores, which are favorable for Cd^2+^ adsorption.

The influence of ionic strength on adsorption can be used to determine the adsorption type for Cd^2+^. The impact of ionic strength on the adsorption of Cd^2+^ by RH and RH-COF was investigated and the results are presented in Figure 6b. The effects of ionic strength varied between the two materials. As ionic strength increased, the Cd^2+^ adsorption capacity of RH was drastically decreased by 52.80–65.62%, indicating that Cd^2+^ was primarily adsorbed on the RH surface as outer-sphere complexes, which was nonspecific adsorption. However, the reduction in adsorption of Cd^2+^ by RH-COF was relatively modest, ranging from 1.68% to10.66% with rising Na^+^ concentration. This indicates that Na^+^ competes with Cd^2+^ for available adsorption sites in this context. The diverse and abundant functional groups on RH-COF facilitated heavy metal complexation or coordination; consequently, the influence of Na^+^ on Cd^2+^ adsorption of RH-COF was weaker than that of RH.

**Figure 7:**
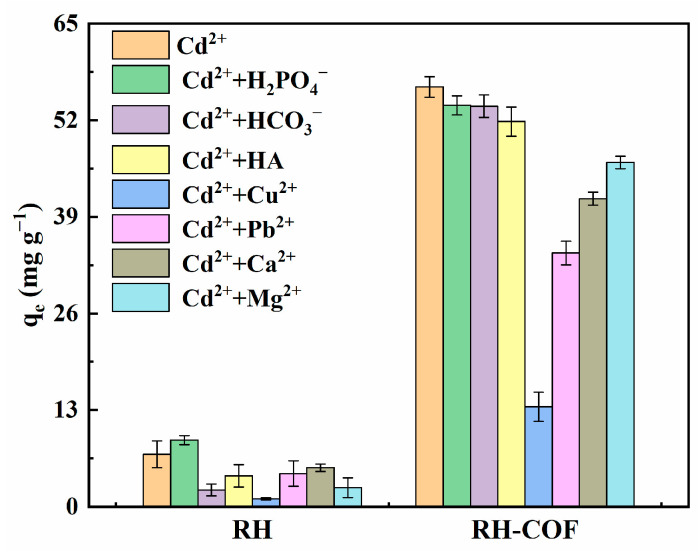
Effect of coexisting ions on the adsorption of Cd^2+^ by RH and RH-COF (initial concentration: 250 mg L^−1^ Cd^2+^, 10^−4^ mol L^−1^ H_2_PO_4_^−^, 10^−3^ mol L^−1^ HCO_3_^−^, 100 mg L^−1^ Cu^2+^, 100 mg L^−1^ Pb^2+^, 10^−2^ mol L^−1^ Ca^2+^, 10^−2^ mol L^−1^ Mg^2+^, and 10^−3^ mol L^−1^ HA, adsorbent dosage: 2.0 g L^−1^, contact time: 48 h).

The influences of coexisting ions, including H_2_PO_4_^−^, HCO_3_^−^, Cu^2+^, Pb^2+^, Ca^2+^, Mg^2+^, and HA, on the adsorption of Cd^2+^ by RH and RH-COF were investigated, with results illustrated in Figure 7. The effects of coexisting ions and HA on Cd^2+^ adsorption were basically in the order of Cu^2+^ > Pb^2+^ > Ca^2+^ > Mg^2+^ > HA, H_2_PO_4_^−^, HCO_3_^−^. It was found that H_2_PO_4_^−^, HCO_3_^−^, and HA exert a minimal effect on the adsorption of Cd^2+^ on RH-COF. Conversely, the adsorption capacity of Cd^2+^ was significantly decreased in the presence of Cu^2+^, Pb^2+^, Ca^2+^, and Mg^2+^. For example, the adsorption capacity of Cd^2+^ decreased to 13.44 mg g^−1^ and 34.15 mg g^−1^ in the presence of Cu^2+^ and Pb^2+^, respectively, suggesting a stronger sorption affinity of Cu^2+^ and Pb^2+^ towards RH-COF. Previous studies demonstrated that certain heavy metals can efficiently inhibit the adsorption of Cd^2+^ due to competition for available binding sites [52,53].

### 3.3. Possible Adsorption Mechanism

In this study, a series of advanced characteristic techniques, such as SEM, EDS, FT-IR, XPS, etc., were used while combining the analysis of surface functional groups and heavy metal chemical forms to elucidate the possible mechanisms of Cd^2+^ adsorption on RH and new RH-COF material.

**Figure 8:**
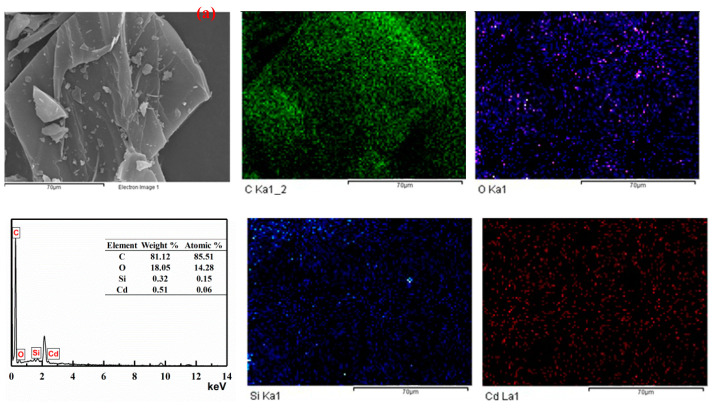

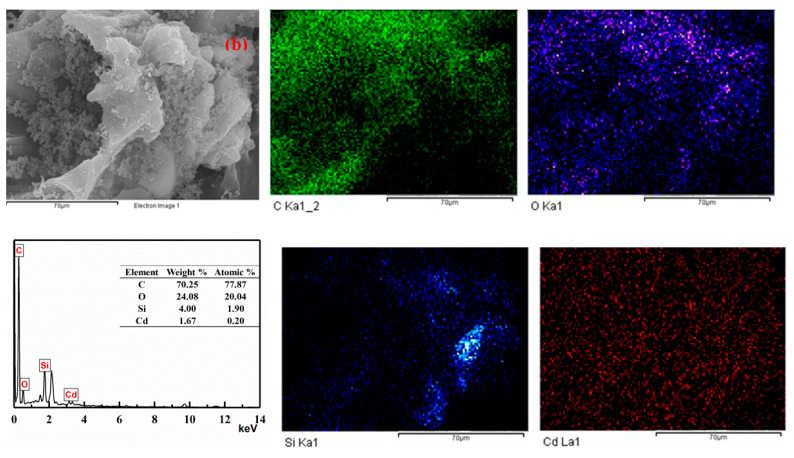
SEM-EDS spectra and SEM mappings of RH (**a**), and RH-COF (**b**), after the adsorption of Cd^2+^.

The SEM-EDS spectra of RH and RH-COF after the adsorption of Cd^2+^ were thoroughly analyzed. The results (Figure 8) showed that the characteristic peaks of Cd appeared in the EDS spectra, confirming successful adsorption of Cd^2+^ on the surface of the adsorbents. Furthermore, SEM mapping was conducted on RH and RH-COF following the adsorption of Cd^2+^ to investigate the final form of the resultant products. Figure 8 verifies that Cd was effectively adsorbed by RH and RH-COF. Notably, the distribution of Cd in the mapping diagram was relatively uniform and closely coincides with that of O. This observation suggests that the oxygen-containing functional groups present on both RH and RH-COF may significantly facilitate the adsorption of Cd^2+^.

**Figure 9:**
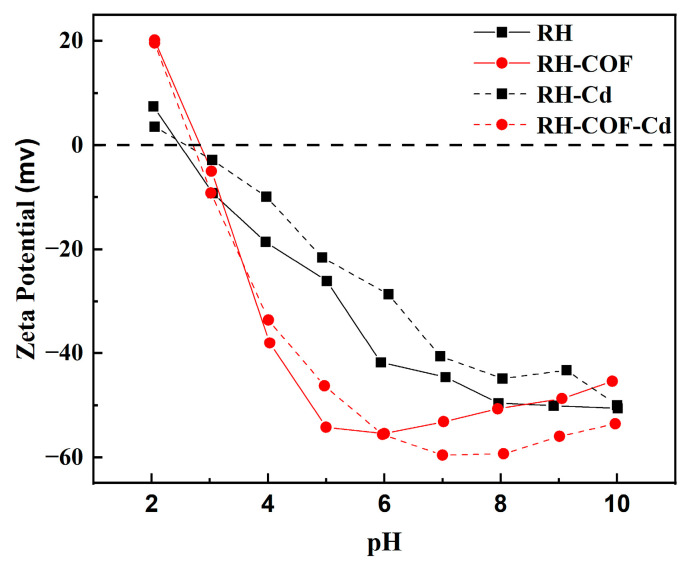
Zeta potential of RH and RH-COF before and after the adsorption of Cd^2+^.

The pHpzc values of RH and RH-COF were relatively low (Figure S1), resulting in the surface of both adsorbents being negatively charged in aqueous solutions, which facilitated the adsorption of Cd^2+^. When the pH exceeded the pHpzc, deprotonation occurred on the surface of RH and RH-COF, allowing for electrostatic interactions that promote Cd^2+^ adsorption. No significant change in the zeta potential was observed for either adsorbent before or after adsorption, suggesting that electrostatic adsorption plays a role in the process, but its contribution is somewhat limited (Figure 9). Within a pH range of 3 to 8, the zeta potential of RH-COF was lower than that of RH, suggesting a significantly higher density of negative charges on the surface of RH-COF compared to RH, so the electrostatic interactions between RH-COF and Cd^2+^ were stronger. As illustrated in Figure 6a, when the pH exceeded 3, the capacity of Cd^2+^ adsorption by RH-COH surpassed that by RH. Furthermore, the equilibrium pH following Cd^2+^ adsorption on RH-COF is lower than that on RH, which indicates a higher concentration of H^+^ in solution without occupying additional adsorption sites on the surface of RH-COF, consequently, more Cd^2+^ bonded with it by electrostatic interactions (Figure S2).

**Figure 10:**
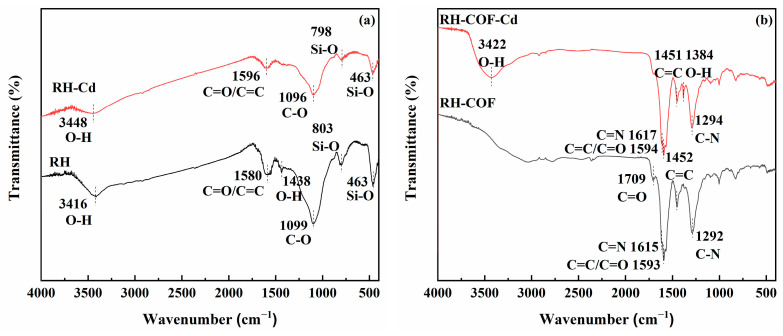
FT-IR spectra of RH (**a**) and RH-COF (**b**) before and after the adsorption of Cd^2+^.

The FT-IR spectra of RH and RH-COF, both prior to and following the adsorption of Cd^2+^, were recorded, with the results presented in Figure 10. After the adsorption of Cd^2+^, the peak intensities of O-H (3416 cm^−1^), C=O/C-C (1580 cm^−1^), C-O (1099 cm^−1^), and Si-O (803 cm^−1^ and 463 cm^−1^) were markedly weaker and the peak of O-H (1438 cm^−1^) disappeared compared with RH (Figure 10a). After the adsorption of Cd^2+^, a reduction in the peak intensities for C=C/C=O (1593 cm^−1^), C=C (1452 cm^−1^), and C-N (1292 cm^−1^) was observed along with a slight shift in their positions. The disappearance of the C=O peak at 1709 cm^−1^ was noted, along with new peaks of O-H (1384 cm^−1^ and 3422 cm^−1^) emerging relative to RH-COF (Figure 10b). These findings indicated that carboxyl and hydroxyl groups might form complexes with Cd^2+^ on the surface of both RH and RH-COF. The absence of the peak corresponding to C=O indicated surface complexation of heavy metals through delocalized π electrons [51,54], which might be the reason for the disappearance of the peak of C=O (1709 cm^−1^) on RH-COF. Following adsorption, it was observed that the peak intensities of Si-O (803 cm^−1^ and 463 cm^−1^) were weaker than those of RH (Figure 10a), indicating that surface complexation between Si-O and Cd^2+^ occurred on the RH surface [45,55].

**Figure 11:**
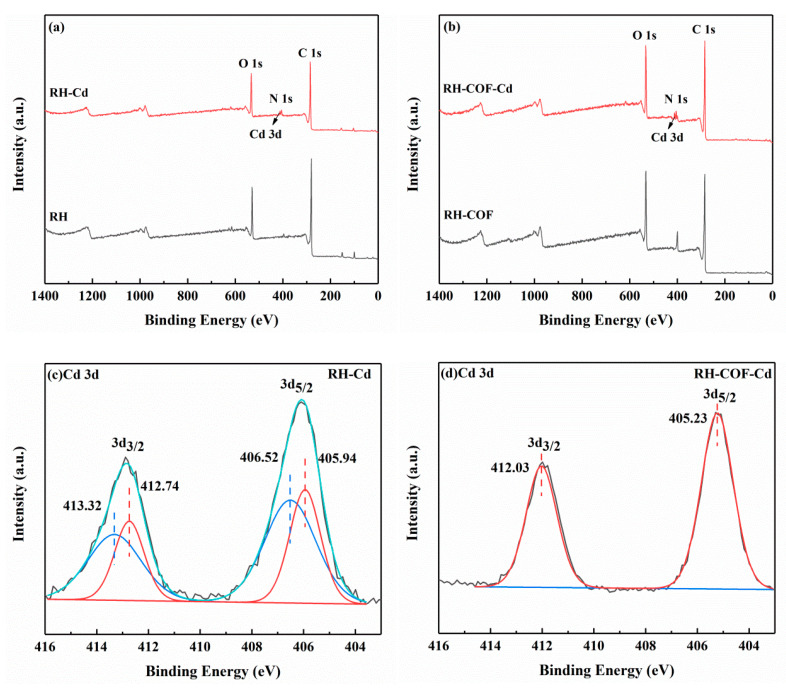

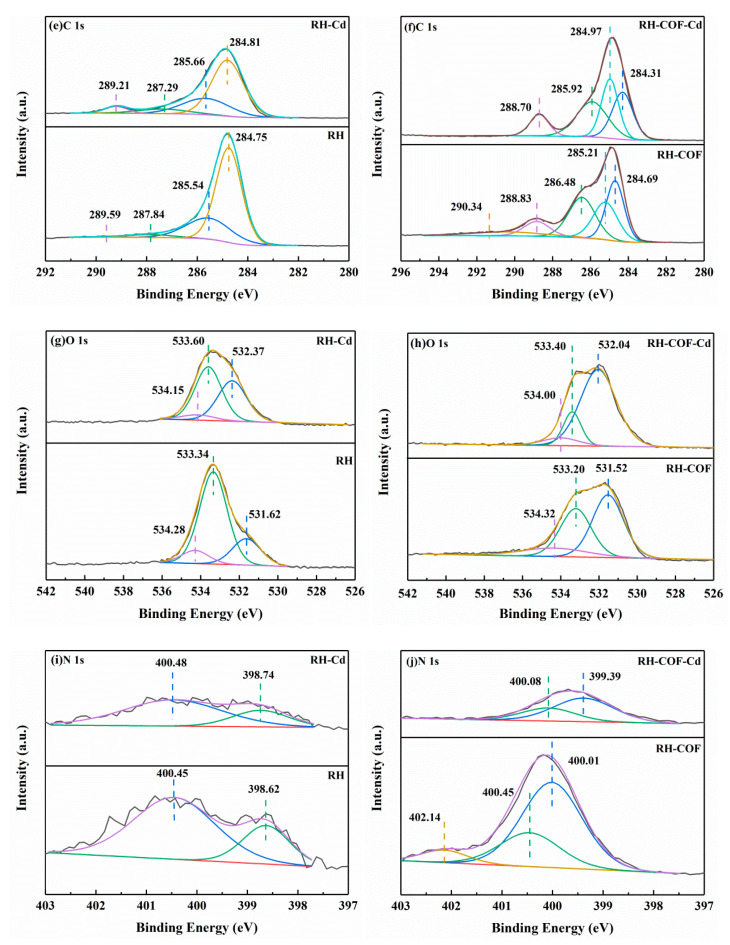
XPS spectra for RH (**a**) and RH-COF (**b**) before and after cadmium uptake, XPS spectra of Cd3d for RH (**c**) and RH-COF (**d**) after cadmium uptake, XPS spectra of C1s for RH (**e**) and RH-COF (**f**) before and after cadmium uptake, XPS spectra of O1s for RH (**g**) and RH-COF (**h**) before and after cadmium uptake, and XPS spectra of N1s for RH (**i**) and RH-COF (**j**) before and after cadmium uptake.

To elucidate the adsorption mechanism of Cd^2+^ by RH and RH-COF, XPS analysis was conducted on RH and RH-COF after Cd^2+^ adsorption, with results presented in Figure 11. The survey spectra of RH and RH-COF after the adsorption of Cd^2+^ showed the presence of new peaks corresponding to Cd 3d, indicating the successful adsorption of Cd^2+^ onto the surface of both materials (Figure 11a,b). Following adsorption, the binding energies for the Cd3d_5/2_ and Cd3d_3/2_ levels in RH were observed at 405.94 eV and 412.74 eV as well as at 406.52 eV and 413.32 eV, respectively (Figure 11c), indicating the formation of CdCO_3_ and Cd(OH)_2_ or Cd-O complexes [56,57,58]. This implied that Cd^2+^ is coordinated to oxygen functional groups (OFGs) on the surface of RH [57,58]. As shown in Figure 11d, the binding energies for Cd3d_5/2_ (405.23 eV) and Cd3d_3/2_ (412.03 eV) levels in RH-COF indicated a similar formation of Cd-O, Cd(OH)_2_, and CdCO_3_ [59,60], alongside the coordination interactions between amino and Cd^2+^ [58], or the chelation involving Cd ion and four ‘O’ [61,62].

Following the adsorption of Cd^2+^, a slight shift was observed in four characteristic peaks of RH in the C1s spectra (Figure 11e), including C-C/C=C (284.75 eV), C-O (285.54 eV), C=O (287.84 eV), and CO_3_^2−^/O=C-O (289.59 eV) [59,63,64,65]. The peak area corresponding to CO_3_^2−^/O=C-O increased from 0.64% to 5.25% (Table S3). This finding indicated that CdCO_3_ precipitation may be formed after Cd^2+^ is adsorbed by RH. Three characteristic peaks of RH were identified in the O1s spectra, including C=O (531.62 eV), C-O/OH (533.34 eV), and O-C=O (534.28 eV) [63,66,67]. After the adsorption of Cd^2+^, the binding energies of the peaks showed a slight shift, and the peak areas of C-O/OH and O-C=O decreased by 27.54% and 3.51%, respectively (Figure 11g) [63,66,67]. This indicated that the hydroxyl and carboxyl groups were involved in the adsorption of Cd^2+^. After Cd^2+^ adsorption of RH-COF, the binding energies of C=O (531.52 eV), C-O/OH (533.20 eV), and O-C=O (534.32 eV) exhibited minor shifts as well (Figure 11h) [63,66,67], concurrently, and the peak areas of C-O/OH and O-C=O decreased from 37.54% and 13.79% to 16.69% and 7.58%, respectively (Table S3). This further suggested that the hydroxyl and carboxyl groups contribute to the adsorption mechanism. At the same time, the peak of O-C=O in the C1s spectra disappeared (Figure 11f), indicating that carboxyl groups play a predominant role in the adsorption of Cd^2+^ by RH-COF.

Following adsorption, the binding energies and the peak area of N1s of RH exhibited no significant changes (Figure 11i), suggesting that the nitrogen-containing functional groups in RH are not involved in the adsorption process. As shown in Figure 11j, three characteristic peaks of RH-COF were identified in the N1s spectra, including -NH_2_ (400.01 eV), C-N (400.45 eV), and C=N (402.14 eV) [67,68,69,70]. After the adsorption of Cd^2+^, the binding energies of the peaks belonging to -NH_2_ and C-N shifted to 399.39 eV and 400.08 eV, respectively, while the peak areas of -NH_2_ and C-N increased by 0.73% and 6.83%, respectively. However, the peak of C=N disappeared. This indicated that the nitrogen-containing functional groups in RH-COF reacted with Cd^2+^, and that amino functional groups may play an important role.

Based on the above results, it can be inferred that carboxyl, hydroxyl, and carbonate functional groups play an important role in the adsorption of Cd^2+^ on RH, primarily forming COO-Cd or Cd-O complexes and CdCO_3_ precipitates. Furthermore, the oxygen-containing and nitrogen-containing functional groups present on the surface of RH-COF enhanced the adsorption capacity of Cd^2+^.

**Figure 12:**
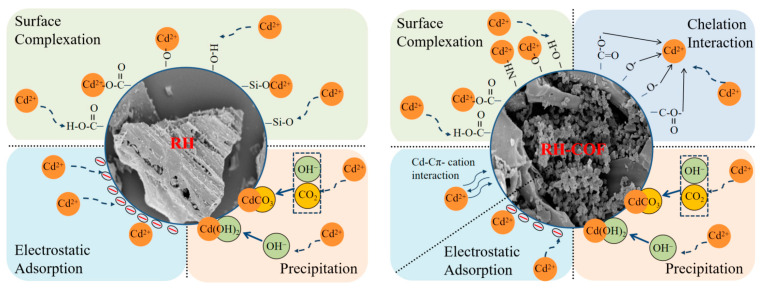
The mechanisms of Cd^2+^ adsorption onto RH and RH-COF.

In summary, the findings from SEM-EDS, zeta potential, FT-IR, and XPS analyses indicated that the potential adsorption mechanisms of Cd^2+^ by RH were dominated by surface complexation and surface precipitation, along with electrostatic adsorption. Conversely, the adsorption of RH-COF on Cd^2+^ was mainly caused by surface complexation and chelation, along with electrostatic adsorption, surface precipitation, and Cπ–cation interactions. The mechanism diagram is illustrated in Figure 12.

## 4. Conclusions

In this work, rice husk biochar was used as the pristine material and a novel covalent organic framework-modified biochar was successfully synthesized via an in-situ polymerization method with Tp and DBd. The resultant COF-modified biochar composite was comprehensively characterized by elemental analysis, BET analysis, SEM, FT-IR, and XPS, confirming the successful modification of the COF on rice husk biochar. In comparison with RH, the pH decreased while the pHpzc value increased for RH-COF. Additionally, there was an enhancement in both the types and quantities of nitrogen- and oxygen-containing functional groups on the surface of RH-COF. The composite demonstrated efficient extraction and preconcentration of Cd^2+^ in aqueous solutions, with a maximum Cd^2+^ adsorption capacity of 58.62 mg g^−1^, which was nearly 14 times greater than that of RH. Furthermore, RH-COF exhibited superior adsorption kinetics compared with RH, indicating that COF modification provided additional active adsorption sites for Cd^2+^. The pseudo-second-order model and the Langmuir model effectively described both the kinetics and isotherms for Cd^2+^ adsorption onto RH-COF, and chemisorption on the monolayer surface played a dominant role in Cd^2+^ removal in this study. Notably, the adsorption performance of Cd^2+^ by RH-COF was hardly influenced by pH values ranging from 4.0 to 8.0. However, ionic strength could regulate the Cd^2+^ adsorption capacity of RH-COF slightly, while certain coexisting ions had more pronounced effects. Mechanistic analysis revealed that the adsorption of Cd^2+^ by RH-COF was dominated by surface complexation and chelation, along with electrostatic adsorption, surface precipitation, and Cπ–cation interactions. Overall, the findings from this study suggest that the synthesis of COF-biochar combined material could be a promising tool for remediation efforts targeting contaminated water.

## Figures and Tables

**Table 1:** Physicochemical properties of materials.

	Sample		
	RH	COF	RH-COF
C (%)	53.34 ± 2.24 a	60.18 ± 1.16 b	36.31 ± 1.20 c
N (%)	0.96 ± 0.03 a	8.88 ± 0.12 b	5.40 ± 0.05 c
O (%)	15.50 ± 2.72 a	22.37 ± 2.88 a	24.08 ± 6.91 a
H (%)	4.01 ± 0.27 a	5.61 ± 0.11 b	4.49 ± 0.09 c
Molar H/C (%)	0.08	0.09	0.12
Molar O/C (%)	0.29	0.37	0.66
Molar (O + N)/C (%)	0.31	0.52	0.81
pH	9.67 ± 0.04 a	7.95 ± 0.04 b	7.90 ± 0.01 b
Bet surface area (m^2^ g^−1^)	2.70	37.37	33.41
Average pore width (nm)	9.24	6.12	6.30
Pore volume (cm^3^ g^−1^)	0.01	0.06	0.05

Different lowercase letters in the table indicate significant differences between treatments at *p* < 0.05.

**Table 2:** The adsorption of Cd^2+^ on other modified biochars.

Adsorbent	pH	Q_m_ (mg g^−1^)	Reference
RH-COF	5.0	58.62	This study
Magnesium oxide–rice husk biochar composite (MgO-BCR)	5.0	18.1	[45]
Calcium-based magnetic biochar (Ca-MBC)	6.0	10.1	[46]
Chitosan–pyromellitic dianhydride modified biochar(CPMB)	5.0	30.12–38.24	[12]
Rice husk	-	7.8	[10]
Modified rice husk	-	8.58–20.24	[47]
RHB/MgAl-layered double hydroxide-coated rice husk(MgAl–LDH@RHB)	6.0	27.46/113.99	[48]
RHB 300, RHB 500, RHB 700/RHB 300-Si, RHB 500-Si, RHB 700-Si	-	52.65, 58.62, 76.55/44.75, 47.83, 60.37	[49]

## Data Availability

Dataset available on request from the authors.

## Supplementary Materials

The following supporting information can be downloaded at https://www.mdpi.com/article/10.3390/toxics12100717/s1, Figure S1: Zeta Potential of RH and RH-COF; Figure S2: Equilibrium pH after adsorption of Cd^2+^ by RH and RH-COF at different initial solution pH; Table S1: Adsorption kinetic parameters of Cd^2+^ on RH and RH-COF (initial concentration of Cd^2+^: 100 mg L^−1^ (RH) and 250 mg L^−1^ (RH-COF), adsorbent dosage: 2.0 g L^−1^, pH 5); Table S2: Isotherm parameters of Langmuir and Freundlich for the adsorption of Cd^2+^ on RH and RH-COF (adsorbent dosage: 2.0 g L^−1^, pH 5); Table S3: The Relative ratio of peaks in C1s, N1s and O1s from XPS.
