# Supplementary material for: Enhanced Adsorption of Cadmium by a Covalent Organic Framework-Modified Biochar in Aqueous Solution
Source: Toxics. 2024 Sep 30;12(10):717. doi: 10.3390/toxics12100717 (PMC11510796; doi:10.3390/toxics12100717)
Supplement: Supplementary file 1 [file toxics-12-00717-s001.zip › toxics-3222506-supplementary.pdf]

Supporting information

Enhanced adsorption of cadmium by a covalent organic framework-modified biochar in aqueous solution

Yanwei Hou [1,2], Shanna Lin [2], Jiajun Fan [2], Youchi Zhang [2], Guohua Jing [1,2,*] and Chao Cai [2,*]

1 College of Chemical Engineering, Huaqiao University, Xiamen 361021, China; houyw@hqu.edu.cn (Y.H.);

2 Key Lab of Urban Environment and Health, Institute of Urban Environment, Chinese Academy of Sciences, Xiamen 361021, China; e-mail@e-mail.com; snlin@iue.ac.cn (S.L.); jjfan@iue.ac.cn (J.F.); yczhang@iue.ac.cn (Y.Z.)

* Correspondence: zhoujing@hqu.edu.cn (G.J.); ccai@iue.ac.cn (C.C.)

Models for kinetics curve fitting

Qseudo-first-order (Eq. (1)) and pseudo-second-order (Eq. (2)) kinetic models were given below

$$ln(q_e - q_t) = lnq_e - k_1 \quad (1)$$

$$\frac{t}{q_t} = \frac{1}{k_2 q_e^2} + \frac{t}{q_e} \quad (2)$$

Where q_e and q_t are the adsorption capacity (mg g^{-1}) at equilibrium and at a given time t (min), respectively; k_1 and k_2 are the rate constant of the pseudo-first-order adsorption (min^{-1}) and the rate constant of pseudo-second-order adsorption (g mg^{-1} min^{-1}), respectively.

Models for isotherm fitting

The Langmuir (Eq. (3)) and Freundlich (Eq. (4)) models were given below

$$Q_e = \frac{Q_m K_L C_e}{1 + K_L C_e} \tag{3}$$

$$Q_e = K_F C_e^{1/n} \tag{4}$$

Where C_e (mg L^{-1}) is the equilibrium concentration of Cd^{2+}; K_L (L mg^{-1}) is the Langmuir constant; Q_m (mg g^{-1}) is the maximum adsorption capacity; K_F (mg g^{-1}) is the constant indicative of the relative adsorption capacity and 1/n is the constant indicative of the intensity of the adsorption.

Table S1 Adsorption kinetic parameters of Cd^{2+} on RH and RH-COF (initial concentration of Cd^{2+}: 100 mg L^{-1} (RH) and 250 mg L^{-1} (RH-COF), adsorbent dosage: 2.0 g L^{-1}, pH 5)

kinetic model	Parameters	RH	RH-COF
pseudo-first-order kinetic model	q_{e1} (mg g^{-1})	3.04	52.27
	k_1 (h^{-1})	0.97	21.02
	R^2	0.77	0.69
pseudo-second-order kinetic model	q_{e2} (mg g^{-1})	3.13	53.08
	k_2 (g mg^{-1} h^{-1})	0.66	0.94
	R^2	0.92	0.90

Table S2 Isotherm parameters of Langmuir and Freundlich for the adsorption of Cd^{2+} on RH and RH-COF (adsorbent dosage: 2.0 g L^{-1}, pH 5)

Isotherm	Parameters	RH	RH-COF
Langmuir	Q_m (mg g^{-1})	4.20	58.62
	K_L (L mg^{-1})	0.19	4.56
	R^2	0.92	0.90
Frendlich	K_F (mg g^{-1})	1.77	46.54
	1/n	0.16	0.05

	R^2	0.83	0.84

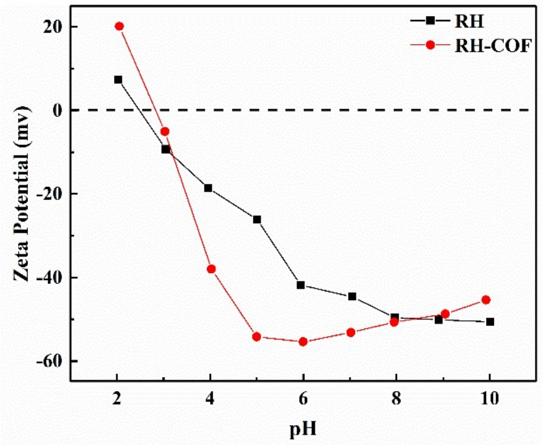

Figure S1 Zeta Potential of RH and RH-COF

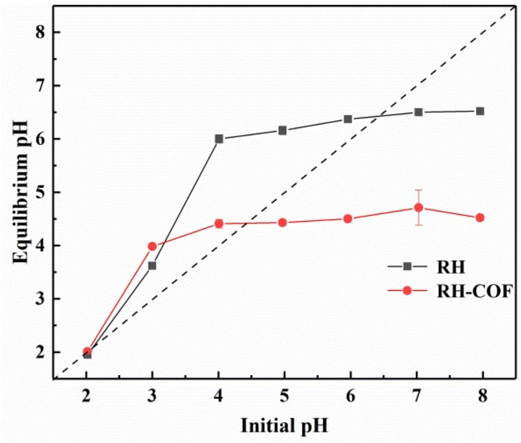

Figure S2 Equilibrium pH after adsorption of Cd^{2+} by RH and RH-COF at different initial solution pH

Table S3 The Relative ratio of peaks in C1s, N1s and O1s from XPS

	Assignment	Sample			
		RH	RH-Cd	RH-COF	RH-COF-Cd
C1s	C-C/C=C	68.65	60.85	28.99	28.01

(%)	C-O	27.02	26.26	24.62	28.84
	C-N	-	-	28.86	31.52
	C=O	3.69	7.84	10.1	11.62
	CO_3^{2-}/O=C-O	0.64	5.25	7.43	-
O1s (%)	C=O	20.69	51.75	48.68	75.73
	C-O/OH	69.50	41.96	37.54	16.69
	O-C=O	9.81	6.30	13.79	7.58
N1s (%)	$-NH_2$	26.33	26.42	66.16	66.89
	C-N	73.67	73.58	26.28	33.11
	-C=N-	-	-	7.55	-
